# Mn_2_O_3_ Nanoparticles as a Potential Neuronal Threat Despite Hepatorenal Benefits—Implications for Dietary Supplementation

**DOI:** 10.3390/antiox15050567

**Published:** 2026-04-29

**Authors:** Karolina Różaniecka-Zwolińska, Ewelina Cholewińska, Przemysław Sołek, Jerzy Juśkiewicz, Katarzyna Ognik

**Affiliations:** 1 Department of Biochemistry and Toxicology, University of Life Sciences, Akademicka 13, 20-950 Lublin, Poland; karolina.rozaniecka@up.edu.pl (K.R.-Z.); ewelina.cholewinska@up.edu.pl (E.C.); przemyslaw.solek@up.edu.pl (P.S.); 2 InLife Institute of Animal Reproduction and Food Research, Polish Academy of Sciences, Trylińskiego 18, 10-683 Olsztyn, Poland; j.juskiewicz@pan.olsztyn.pl

**Keywords:** manganese nanoparticles, oxidative stress, neurotoxicity, antioxidant defense, brain damage, dietary supplementation, Mn_2_O_3_, hepatorenal protection, protein nitration, rats

## Abstract

Manganese (Mn) is an essential trace element crucial for antioxidant defense, metabolism, and neuronal function, yet both deficiency and excess may induce oxidative stress and organ-specific damage. This study investigated the effects of dietary manganese exclusion and replacement of standard MnCO_3_ with Mn_2_O_3_ nanoparticles on redox status and oxidative damage in rats. Twenty-four male Wistar rats were divided into three groups: control (K) receiving 65 mg/kg Mn as MnCO_3_, manganese-deficient (B), and nanoparticle-supplemented (N) receiving 65 mg/kg Mn as Mn_2_O_3_ nanoparticles. After 12 weeks, tissues were analyzed for oxidative stress markers and antioxidant enzyme activities. Manganese deficiency resulted in decreased plasma SOD activity, increased lipid peroxidation, and severe oxidative–nitrosative damage in the brain and jejunum, despite hepatic compensatory mechanisms. Mn_2_O_3_ nanoparticle supplementation enhanced hepatic and renal antioxidant capacity, reducing oxidative damage in these organs. However, nanoparticles induced pronounced neurotoxicity, characterized by GSH depletion, elevated DNA damage (8-OHdG), protein nitration (3-NT), and caspase activation in brain tissue. These findings demonstrate that while Mn_2_O_3_ nanoparticles offer improved bioavailability and hepatorenal benefits, they pose significant neurotoxic risks, necessitating caution in dietary supplementation strategies.

## 1. Introduction

Manganese (Mn) is an inorganic trace element involved in numerous physiological processes in living organisms. It plays a crucial role in proper growth and development, antioxidant defense, reproduction, regulation of blood glucose levels and blood coagulation, protection against reactive oxygen species, immune defense, and neuronal and digestive functions [1,2,3,4,5,6,7].

Manganese participates in biochemical reactions as a component or activator of enzymes that form metal–enzyme complexes, including pyruvate carboxylase, manganese superoxide dismutase (Mn-SOD), glutamine synthetase, glycosyltransferase, alkaline phosphatase, and arginase, thereby playing a key role in their activity regulation [4,8,9,10,11]. This element is also critically involved in metabolism, including amino acids, lipids, proteins, and carbohydrates [12,13]. Moreover, manganese has a significant impact on bone regeneration, mineralization, and skeletal strength by actively participating in bone remodeling processes [14].

To ensure the proper course of these physiological processes, a constant dietary supply of Mn is essential in animals. Therefore, laboratory animal diets are supplemented with mineral mixtures containing Mn in amounts consistent with dietary recommendations. Manganese deficiency in the diet may result in increased mortality, growth retardation, reduced appetite, anemia, decreased blood cholesterol levels, thyroid enlargement, cataracts, reproductive system dysfunction, and reduced Mn content in tissues [3,4,15]. Severe Mn deficiency can lead to skeletal developmental abnormalities and even serious congenital defects and seizures [16].

However, elevated concentrations of manganese in the body may contribute to adverse physiological effects, such as oxidative stress, alterations in physiological parameters, and impairment of multiple organ systems, potentially leading to death [17]. Excessive Mn levels may disrupt oxidative phosphorylation and increase the production of reactive oxygen species (ROS), resulting in excessive ROS accumulation and redox imbalance, ultimately causing oxidative stress (OS) [17]. Oxidative stress is one of the recognized mechanisms of manganese toxicity. High OS levels may affect nitration processes, leading to direct damage to proteins, amino acids or nucleic acids, and consequently to cellular structural abnormalities and cell death [18].

Research findings regarding the effect of manganese on immune function are inconclusive. Long-term exposure to manganese compounds, such as manganese chloride, has been shown to significantly enhance the humoral immune response in rodents, while manganese is also capable of improving cellular immunity and promoting the growth and development of immune organs [17]. In contrast, excessive manganese exposure suppresses both humoral and cellular immune responses [19].

As a trace element, manganese accumulates in tissues and organs, and its concentration at different levels depends primarily on dose, chemical form, physiological factors, and duration of exposure [19,20]. Exposure to Mn^2+^ induces mild hepatic congestion, vacuolar degeneration of hepatocytes, increased lipid peroxidation, and reduced antioxidant capacity in rats. Chronic exposure to Mn^2+^ may result in manganese bioaccumulation in the liver and subsequent hepatic damage [21].

Due to the physiological functions of manganese, animal diets are commonly supplemented with this element to meet nutritional requirements. Although manganese is nutritionally essential at low concentrations, it becomes potentially toxic at higher levels. Excessive manganese exposure may be associated with hepatotoxicity, neurotoxicity, and reproductive dysfunction [22]. Manganese accumulated in excess primarily causes toxic effects and neurological disorders known as manganism, particularly affecting brain structures. These disorders may manifest as behavioral changes, irritability, aggressiveness, slow and uncoordinated movements, tremors, gait disturbances, dementia, muscle spasms, and hallucinations. The pathophysiology of manganism is complex; however, it has been demonstrated that Mn_2_O_3_ nanoparticles may penetrate the blood–brain barrier, as evidenced by their small size (<100 nm) and surface properties known to facilitate CNS entry, potentially leading to neuronal accumulation [23,24,25].

Nanoparticles represent a promising alternative to traditional forms of trace elements. Data indicate that nanoparticles may induce moderate ROS-generating and inflammatory responses [26] and may also trigger carcinogenic processes, including protein, lipid, and DNA damage, as well as epigenetic alterations [27]. However, numerous studies demonstrate that the small size of nanoparticles enhances their biological activity, allowing for a reduction in the number of nanoparticles added to diets compared to conventional macro-sized mineral forms [28]. Due to their high physicochemical reactivity, manganese nanoparticles may be used as an alternative and effective promoter of health and growth at much lower doses than inorganic Mn forms, thereby significantly reducing manganese excretion into the environment [28]. Given that manganese is an essential trace element but excessive concentrations may induce oxidative stress and health disturbances, there is a need to develop more effective and safer strategies for its supplementation. Male Wistar rats were selected as they represent an established model for nanotoxicology and trace mineral nutrition studies, exhibiting comparable Mn metabolism and oxidative stress responses to humans. This outbred strain provides robust physiological relevance for assessing nanoparticle bioavailability and safety in dietary applications.

This study aimed to determine the effect of manganese exclusion from the dietary mineral mixture and the dietary replacement of the recommended level of MnCO_3_ with Mn_2_O_3_ nanoparticles (Mn_2_O_3_NPs) on redox status, protein nitration and DNA methylation in rats.

## 2. Materials and Methods

The present study is part of a broader research framework focused on the diverse biological effects associated with manganese deficiency and supplementation with manganese (III) oxide nanoparticles. The experimental design, as well as the applied methodologies and procedural aspects, has been described in detail in earlier publications by Cholewińska et al. [29,30], Sołek et al. [31], and Różaniecka-Zwolińska et al. [32].

### 2.1. Animals and Housing Conditions

All procedures involving animals were conducted in strict accordance with the national regulations on animal experimentation in the Republic of Poland and were reviewed by an ethics committee in compliance with the European Convention for the Protection of Vertebrate Animals Used for Experimental and Other Scientific Purposes, as well as Directive 2010/63/EU [33]. The study protocol received formal approval from the relevant Local Institutional Animal Care and Use Committee (Approval No. 13/2022, Olsztyn, Poland, 16 March 2022). To minimize pain, suffering, and distress, animals were housed individually to prevent aggression, maintained under standardized environmental conditions, monitored daily for welfare, anesthetized before blood collection and sacrifice, and subjected to predefined humane endpoints.

Twenty-four animals meeting the inclusion criteria, i.e., clinically healthy male Wistar albino rats (outbreds Cmdb:WI, Białystok, Poland) aged four weeks, were randomly assigned to three experimental groups (n = 8 in each group). Animals were obtained from the breeding unit of the Institute of Animal Reproduction and Food Research PAS (Olsztyn, Poland; breeder registry No. 051). The sample size (n = 8 per group) was determined a priori based on a power analysis of the primary outcomes conducted in G*Power (Heinrich-Heine-Universität Düsseldorf, Düsseldorf, Germany, version 3.1.9.7). The calculation assumed a significance level of α = 0.05 and statistical power (1 − β) = 0.80. The expected effect size (Cohen’s f = 0.30) was derived from our previous studies using the same experimental model and endpoints. This analysis indicated that a minimum of 7 animals per group was required; therefore, a sample size of 8 per group was selected to ensure sufficient power while remaining consistent with the 3R principles. Allocation to groups was randomized by generating numbers with MS Excel’s RAND() function (Microsoft Corporation, Redmond, WA, USA, version 2511). To minimize environmental bias, cages were positioned to ensure equivalent rack locations (top, bottom, left, right) across treatment groups. Additionally, cage locations were arranged to balance representation across all rack positions, and analytical personnel remained blinded to group allocations. Only the project manager had complete knowledge of individual animals’ treatment group assignments. The animals were housed individually in stainless steel cages under controlled environmental conditions, including a constant temperature of 21–22 °C, relative humidity maintained at 60 ± 10%, a 12 h light/dark cycle, and an air exchange rate of 15 changes per hour. Throughout the 12-week experimental period, the rats had unrestricted access to tap water and semi-purified diets. The diets were freshly prepared, stored in airtight containers at 4 °C, and used until the completion of the study (details in Table 1).

**Table 1:** Composition of basal experimental diet fed to rats, %.

Ingredient	Content
Unchangeable ingredients:	
Casein ^1^	14.8
DL-methionine	0.2
Cellulose ^2^	8.0
Choline chloride	0.2
Rapeseed oil	8.0
Cholesterol	0.3
Vitamin mix ^3^	1.0
Maize starch ^4^	64.0
Changeable ingredient:	
Mineral mix (MX) ^5^	3.5
Calculated content:	
Crude protein	13.5

^1^ Casein preparation: crude protein 89.7%, crude fat 0.3%, ash 2.0%, and water 8.0%. ^2^ α-Cellulose (SIGMA, Poznan, Poland), main source of dietary fiber. ^3^ AIN-93G-VM [34], g/kg mix: 3.0 nicotinic acid, 1.6 Ca pantothenate, 0.7 pyridoxine-HCl, 0.6 thiamin-HCl, 0.6 riboflavin, 0.2 folic acid, 0.02 biotin, 2.5 vitamin B-12 (cyanocobalamin, 0.1% in mannitol), 15.0 vitamin E (all-rac-α-tocopheryl acetate, 500 IU/g), 0.8 vitamin A (all-trans-retinyl palmitate, 500,000 IU/g), 0.25 vitamin D-3 (cholecalciferol, 400,000 IU/g), 0.075 vitamin K-1 (phylloquinone), 974.655 powdered sucrose. ^4^ Maize starch preparation: crude protein 0.6%, crude fat 0.9%, ash 0.2%, total dietary fiber 0%, and water 8.8%. ^5^ Changeable dietary ingredient in relation to manganese level; mineral mixture (the base according to NRC, [35]) with standard Mn level and deprived of Mn, see Table 2 and Table 3.

**Table 2:** Experimental schema * (provided manganese dosage was calculated taking into account MnCO_3_ in MX or Mn from Mn_2_O_3_ nanoparticles preparation).

Group	12 Weeks of Feeding
B (Negative CONT, without Mn in MX)	A diet with MX deprived of Mn (n = 8)
K (Control, with standard supplementation of Mn in MX)	A diet containing 65 mg/kg Mn from MnCO_3_ (n = 8)
N (Nano Mn, with standard supplementation of Mn but from novel nanoparticle source)	A diet containing 65 mg/kg Mn from Mn_2_O_3_ nanoparticles (n = 8)

n = 8, number of rats used in a particular feeding period. * Experimental groups: B—during all twelve weeks of feeding, the Mn-deficient rats were given a diet with MX deprived of Mn (MnCO_3_ excluded from MX); K—the rats were fed a diet with standard mineral mixture (MX) resulting in 65 mg Mn (from MnCO_3_ in MX) per 1 kg of a diet during 12 weeks of feeding; N—the rats were given a diet containing 65 mg/kg Mn from Mn_2_O_3_ nanoparticles preparation per 1 kg of a diet during 12 weeks of feeding.

**Table 3:** Composition of mineral mixtures (MXs) used in experimental diets.

	MX with Standard Mn Dosage ^1^	MX Deprived of Mn ^2^
Calcium carbonate anhydrous CaCO_3_	357	357
Potassium phosphate monobasic K_2_HPO_4_	196	196
Potassium citrate C_6_H_5_K_3_O_7_	70.78	70.78
Sodium chloride NaCl	74	74
Potassium sulfate K_2_SO_4_	46.6	46.6
Magnesium oxide MgO	24	24
Microelement mixture:	18	18
Starch	To 1000 g = 213.62	To 1000 g = 213.62
Microelement mixture:		
Ferric citrate [16.7% Fe]	31	31
Zinc carbonate ZnCO_3_ [56% Zn]	4.5	4.5
Manganous carbonate MnCO_3_ [44.4% Mn]	23.4	0
Copper carbonate CuCO_3_ [55.5% Cu]	1.85	1.85
Potassium iodate KJ	0.04	0.04
Citric acid C_6_H_8_O_7_	To 100 g = 39.21 g	To 100 g = 62.61

^1^ given to K group (12 weeks of feeding), ^2^ given to B and N groups (12 weeks of feeding), but the N group was provided with the appropriate amount of Mn from the Mn_2_O_3_ nanoparticle preparation as an emulsion along with dietary rapeseed oil.

### 2.2. Experimental Diets and Study Design

The experimental diets were based on a modified casein formulation for laboratory rodents in accordance with recommendations issued by the American Institute of Nutrition. Three dietary variants were designed to assess the impact of different forms of manganese supplementation. The control group (K) received a diet supplemented with 65 mg/kg Mn derived from the standard mineral mixture. The negative control group (B) was provided with a diet in which Mn was omitted from the mineral mixture. In contrast, group N was fed a diet containing 65 mg/kg Mn supplied in the form of manganese oxide nanoparticles (Mn_2_O_3_-NPs). The Mn_2_O_3_ nanoparticles were purchased from Sky Spring Nanomaterials Inc. (Houston, TX, USA). According to the manufacturer’s specifications, the material was characterized by high purity (99.9%) and well-defined physicochemical properties, including a particle size range of 40–60 nm, density of 7.3 g/cm^3^, melting point of 1519 K, and boiling point of 2334 K. These parameters were considered suitable for ensuring stability and repeatability in nutritional experiments. For safety and technological reasons, the nanoparticles were not incorporated directly into the mineral premix but were administered separately in the oil phase, which minimized the risk of airborne exposure and improved distribution within the diet. For this purpose, prior to incorporation, the nanoparticles were dispersed in a calculated volume of rapeseed oil serving as a compatible carrier, and the suspension was subjected to sonication to enhance dispersion and limit particle aggregation. The resulting emulsion was then added to the diet after thorough mixing of the basal components and mineral mixtures. The precise composition of the mineral mixtures used across all experimental groups is presented in Table 2 and Table 3. Additionally, the hydrodynamic size distribution and zeta potential of the Mn_2_O_3_ nanoparticles, determined using dynamic light scattering (DLS) with a Zetasizer Nano ZS (Malvern Instruments, Malvern, UK), have been described in our previous study [3].

### 2.3. Sample Collection and Tissue Preparation

No formal a priori exclusion criteria were established beyond standard welfare monitoring. Animals showing prolonged food refusal (>2 days), distress, neurological deficits, or blood in feces (>24 h) would have been humanely euthanized. No exclusions or adverse events occurred, and all 24 rats (n = 8 per group: B, K, N) completed the 12-week study with normal body weight gain and welfare, and all samples were analyzed (n = 8 per group). At the end of the 12-week feeding period, the animals were sacrificed and biological material was collected for further analyses. Blood samples were obtained from the caudal vena cava of anesthetized animals (n = 8 per group). Anesthesia was administered through intraperitoneal injection of ketamine and xylazine at dosages of 100 mg/kg BW and 10 mg/kg BW, respectively. After blood collection, the euthanasia was conducted via dislocation of the cervical vertebrae. Blood was drawn into heparinized tubes and centrifuged to obtain plasma. Immediately after dissection, the liver, kidneys, jejunum, and brain fragments were excised, rinsed in cold physiological saline, weighed and homogenized (n = 8 per group). The homogenates were subsequently centrifuged and the resulting supernatants were used for biochemical determinations. Protein concentration in tissue homogenates was determined using the BCA method to enable normalization of results.

### 2.4. Biochemical Analyses

In both plasma and tissue homogenates, parameters associated with oxidative stress, antioxidant defense, protein oxidation, protein nitration, apoptotic activity, and oxidative DNA damage (n = 8 per group for all) were evaluated using commercially available ELISA-based immunoassay kits, according to the manufacturers’ instructions: malondialdehyde (MDA; MBS268427; MyBioSource Inc., San Diego, CA, USA), 3 nitrotyrosine (3-NT; MBS284212; MyBioSource Inc., San Diego, CA, USA), 8 hydroxydeoxyguanosine (8-OHdG; MBS267513; MyBioSource Inc., San Diego, CA, USA), caspase 3 (Casp-3, MBS261814; MyBioSource Inc., San Diego, CA, USA), caspase 8 (Casp-8, MBS260539; MyBioSource Inc., San Diego, CA, USA), total antioxidant capacity (TAC, MBS2548432; MyBioSource Inc., San Diego, CA, USA), total glutathione (TGSH; EIAGSHC; Thermo Fisher Scientific, Waltham, MA, USA), oxidized glutathione (GSSG; EIAGSHC; Thermo Fisher Scientific, Waltham, MA, USA), reduced glutathione (GSH; EIAGSHC; Thermo Fisher Scientific, Waltham, MA, USA), carbonyl protein (PC; E-BC-K117-S; Elabscience, Houston, TX, USA). Absorbance was measured with a microplate reader (Sunrise™ microplate reader, Tecan Group Ltd., Männedorf, Switzerland) and concentrations were calculated from standard calibration curves. In plasma, superoxide dismutase (SOD in plasma MBS3809923, SOD in tissues homogenates MBS036924; MyBioSource Inc., San Diego, CA, USA) and catalase (CAT in plasma MBS726781, CAT in tissues homogenates MBS701908; MyBioSource Inc., San Diego, CA, USA) were determined as protein concentrations (enzyme concentration), whereas in tissue homogenates their enzymatic activities were measured. Additionally, plasma concentrations of selected mineral elements, Fe (No. Cat.3-334, PZ Cormay S.A., Łomianki, Poland), Cu (No. Cat. 993305, Biomaxima S.A., Lublin, Poland), and Zn (No. Cat. 992814, Biomaxima S.A., Lublin, Poland), were assessed using appropriate commercial diagnostic assays (n = 8 per group for all).

### 2.5. Global DNA Methylation Analysis

Global DNA methylation was determined as the percentage of 5-methylcytosine (5-mC) in genomic DNA (n = 8 per group for all tested tissues) isolated from the collected biological material using standard extraction procedures. The level of DNA methylation was quantified with the MethylFlash Global DNA Methylation (5-mC) ELISA Easy Kit (Colorimetric; P-1030-96) from EpigenTek Group Inc. (Farmingdale, NY, USA), following the manufacturer’s instructions. Absorbance was read with a microplate reader (Sunrise™ microplate reader, Tecan Group Ltd., Männedorf, Switzerland) and the percentage of methylated cytosines was calculated relative to the standards provided with the assay.

### 2.6. Statistical Analysis

Results were expressed as mean values with standard error of the mean (SEM). Statistical significance of differences between the experimental groups (K, N and B; n = 8 per group) was assessed using one-way analysis of variance (ANOVA) using Statistica software version 14.1.0.4. (TIBCO Software Inc., Tulsa, OK, USA). When significant effects were observed, differences among group means were evaluated using Duncan’s multiple range test. This post hoc method was chosen for its sensitivity in detecting diet-related differences across multiple intervention groups. Prior to analysis, data were examined to ensure that underlying statistical assumptions were met. Normality of residuals was assessed using the Shapiro–Wilk test, while homogeneity of variances was evaluated with the Brown–Forsythe test. Statistical significance was defined as a *p*-value < 0.05.

## 3. Results

Complete exclusion of Mn from the mineral mixture used in the diet of rats resulted in a decrease in plasma SOD and PC levels (*p* = 0.005 and *p* = 0.001, respectively), accompanied by an increase in MDA and CASP-8 levels (*p* = 0.002 and *p* < 0.001, respectively), compared with the control group receiving the recommended dietary level of Mn in the standard MnCO_3_ form (Table 4).

In the livers of rats fed a Mn-deficient diet, lower levels of 8-OHdG and 3-NT were observed (*p* = 0.004 and *p* < 0.001, respectively), along with higher SOD activity (*p* < 0.001) and increased GSH and PC contents (*p* = 0.016 and *p* = 0.005, respectively), compared with the control group (Table 5).

This dietary intervention also led to a reduction in CAT activity (*p* = 0.002) and 8-OHdG levels (*p* < 0.001) in the kidneys of the examined rats (Table 6).

In the jejunum of rats fed a Mn-deficient diet, increased SOD and CAT activities (*p* < 0.001 for both) as well as elevated levels of MDA (*p* = 0.003), CASP-3, 3-NT, and PC (*p* < 0.001 for all) were noted. In contrast, in the brain, decreased MDA and GSH contents (*p* < 0.001 for both) and reduced PC levels (*p* = 0.006) were observed, accompanied by increased levels of 8-OHdG, CASP-3, CASP-8, and 3-NT (*p* < 0.001, *p* = 0.014, *p* = 0.002, and *p* = 0.027, respectively; Table 7 and Table 8).

Replacement of the standard Mn form (MnCO_3_) with Mn_2_O_3_ nanoparticles in the mineral mixture added to the rat diet resulted in decreased plasma SOD and Cu levels (*p* = 0.005 and *p* = 0.006, respectively), while plasma TAS (*p* = 0.013) and CASP-8 content (*p* < 0.001) were increased (Table 4).

In the livers of rats receiving Mn_2_O_3_ nanoparticles in the diet, a reduction in 3-NT levels (*p* < 0.001) was observed, accompanied by increased SOD activity (*p* < 0.001) and PC content (*p* = 0.005), compared with the control group fed a diet containing the standard Mn form (MnCO_3_) (Table 5).

Moreover, substitution of MnCO_3_ with Mn_2_O_3_ nanoparticles in the rat diet resulted in decreased CAT activity (*p* = 0.002) and reduced levels of MDA and 8-OHdG (*p* = 0.002 and *p* < 0.001, respectively) in the kidneys, as well as increased MDA, GSH, and CASP-3 contents in the jejunum (*p* = 0.003, *p* = 0.046, and *p* < 0.001, respectively) (Table 6 and Table 7).

In the brains of rats subjected to this experimental treatment, increased levels of 8-OHdG, CASP-3, CASP-8, and 3-NT were recorded (*p* < 0.001, *p* = 0.014, *p* = 0.002, and *p* = 0.027, respectively), along with a concomitant decrease in GSH and PC contents (*p* < 0.001 and *p* = 0.006, respectively), relative to the control group (Table 8).

Additionally, the study demonstrated higher plasma GSH and Zn levels (*p* = 0.027 and *p* = 0.013, respectively) and lower CASP-8 content in the kidneys (*p* = 0.005) of rats fed a diet supplemented with Mn_2_O_3_ nanoparticles instead of standard MnCO_3_, compared with animals whose diet contained a mineral mixture completely devoid of Mn (Table 4 and Table 6).

## 4. Discussion

Complete exclusion of manganese from the mineral mixture resulted in decreased plasma levels of SOD and PC, accompanied by increased concentrations of MDA and CASP-8 compared with the control group. Manganese, as an essential cofactor of Mn-SOD, is a critical trace element for the neutralization of the mitochondrial superoxide anion [36]. Reduced SOD activity is a direct consequence of cofactor deficiency, leading to a diminished capacity for rapid detoxification of reactive oxygen species (ROS). As a result, susceptibility to oxidative stress increases, along with the risk of cellular damage [37,38].

Elevated MDA concentrations indicate intensified lipid oxidative stress resulting from reduced Mn-SOD activity. The increase in CASP-8, an initiator caspase of apoptosis, suggests that oxidative and mitochondrial stress induced by manganese deficiency activate pro-apoptotic signaling pathways. Proteins remain the primary targets of ROS under Mn-deficient conditions, and their oxidative modification may lead to metabolic disturbances, particularly in metabolically active tissues such as the liver and heart [36].

In the livers of rats fed a Mn-deficient diet, lower levels of 8-OHdG and 3-NT were observed, accompanied by increased SOD activity and higher GSH and PC contents. As the primary organ responsible for manganese storage, the liver activates intensive compensatory mechanisms under conditions of Mn deficiency [39]. Lower 8-OHdG and 3-NT reflect effective genomic protection via upregulated non-Mn-SOD antioxidants (Cu/Zn-SOD, GSH), despite Mn-SOD deficiency. The liver prioritizes DNA integrity over protein protection, as evidenced by elevated PC, due to its high regenerative capacity and metabolic demands. Increased SOD activity and glutathione (GSH) levels protect DNA from oxidative damage, explaining the reduced concentrations of DNA damage (8-OHdG) and nitration (3-NT) markers. This indicates that under Mn deficiency, the liver reprioritizes its antioxidant defense strategy, preserving genomic integrity at the expense of increased protein oxidation, as reflected by elevated PC levels.

In the kidneys of Mn-deficient rats, reduced catalase (CAT) activity and lower 8-OHdG levels were observed. Decreased CAT activity reflects a shift in the renal antioxidant profile. The kidneys rely more heavily on alternative antioxidant systems, such as GSH and glutathione peroxidase (GPx), rather than catalase, which explains the effective protection of DNA despite reduced CAT activity [22,39]. However, long-term consequences may include gradual damage to protein–lipid structures and progressive renal functional impairment [22].

In the jejunum of rats fed a Mn-deficient diet, increased SOD and CAT activities, as well as elevated levels of MDA, CASP-3, 3-NT, and PC, were observed. The upregulation of both antioxidant enzymes indicates an intense mobilization of defense mechanisms in response to severe oxidative stress; however, these responses appear insufficient. Manganese deficiency promotes oxidative stress-mediated damage to tight junction (TJ) proteins, increasing intestinal permeability [40], which in turn enhances susceptibility to inflammatory bowel diseases as well as immunological and metabolic disturbances [33]. This combination of oxidative, nitrosative, and apoptotic stress likely results from direct exposure of the intestinal epithelium to low Mn concentrations in the intestinal lumen and from a local deficiency of Mn-SOD in enterocytes [36,40].

In the brains of Mn-deficient rats, reduced levels of MDA, GSH, and PC were accompanied by increased concentrations of 8-OHdG, CASP-3, CASP-8, and 3-NT. This profile indicates rapid exhaustion of antioxidant defense systems and severe DNA damage. Manganese readily crosses the blood–brain barrier and accumulates in receptor-rich regions such as the basal ganglia and cholinergic system, where it exerts neurotoxic effects [23,25]. Deficiency of Mn-SOD in the brain disrupts mitochondrial neuronal function and neurochemical homeostasis, potentially increasing susceptibility to neurodegenerative diseases and cognitive deficits [32,41]. Chronic intestinal inflammation may further affect brain function via the gut–brain axis [40,42].

Replacement of the standard Mn form (MnCO_3_) with Mn_2_O_3_ nanoparticles reduced plasma SOD and Cu levels while increasing TAS and CASP-8 concentrations. Additionally, higher plasma levels of GSH and Zn were observed. Mn_2_O_3_ nanoparticles may release manganese ions differently within the gastrointestinal tract and may less efficiently replenish the Mn pool available for Mn-SOD. The reduction in plasma SOD activity was compensated by enhanced non-enzymatic antioxidant systems (GSH, TAS), suggesting that Mn_2_O_3_ nanoparticles may reduce ROS generation in plasma, thereby preserving glutathione levels and increasing total antioxidant capacity [31,32]. However, the increase in CASP-8 is concerning and may indicate enhanced apoptosis in peripheral tissues or altered inflammatory signaling pathways.

In the livers of rats receiving Mn_2_O_3_ nanoparticles instead of MnCO_3_, decreased 3-NT levels were observed alongside increased SOD activity and PC content. As the primary manganese storage organ, the liver exhibits superior absorption and utilization of nanoparticles. Enhanced nanoparticle bioavailability, as demonstrated by Cholewińska et al. [29], likely facilitates more efficient manganese delivery to hepatocyte mitochondria, resulting in increased Mn-SOD activity [12,15,43]. The reduction in 3-NT reflects decreased nitrosative stress, which is beneficial for protein integrity.

However, the concomitant increase in PC despite elevated SOD activity suggests that Mn_2_O_3_ nanoparticles may induce additional ROS-generating pathways via Mn^3+^/Mn^2+^ redox cycling or alter mitochondrial metabolism in hepatocytes. This leads to increased oxidative protein modifications even in the presence of enhanced Mn-SOD activity [43,44]. Long-term accumulation of oxidatively modified proteins in the liver may promote inflammation and metabolic dysfunction, with potential consequences for gene expression and metabolic health [3,28]. Nanoparticles have also been shown to disrupt the epigenome through changes in gene expression, histone modifications, and DNA methylation [27].

In the kidneys of rats fed a diet containing Mn_2_O_3_ nanoparticles instead of MnCO_3_, reduced CAT activity, MDA, 8-OHdG, and CASP-8 levels were observed. This profile suggests pronounced renal protection against oxidative stress. Reduced oxidative burden protects both lipids (lower MDA) and DNA (lower 8-OHdG), while decreased CASP-8 indicates reduced renal cell apoptosis. Lower CAT activity reflects a diminished requirement for enzymatic H_2_O_2_ detoxification under conditions in which oxidative stress is better controlled by Mn_2_O_3_ nanoparticles, suggesting greater biocompatibility of nanoparticles compared with MnCO_3_ in renal tissue. Supplementation with Mn_2_O_3_ nanoparticles may therefore protect the kidneys from ROS-induced damage, reducing the risk of chronic inflammation and fibrosis.

In the jejunum of rats fed manganese nanoparticles, increased levels of MDA, GSH, CASP-8, and CASP-3 were observed. Mn_2_O_3_ nanoparticles undergo dissolution in the mildly acidic jejunal environment (pH 6.0–7.0), releasing Mn^3+^ ions that rapidly cycle to Mn^2+^ while catalyzing Fenton-like reactions with H_2_O_2_ to generate highly reactive hydroxyl radicals (- OH). This surface-mediated redox cycling occurs predominantly at nanoparticle-enterocyte interfaces, explaining the localized ROS burst and subsequent lipid peroxidation despite systemic antioxidant compensation [21]. Elevated MDA reflects lipid peroxidative damage in enterocytes, while increased GSH represents a compensatory response involving enhanced synthesis of the primary non-enzymatic antioxidant. The simultaneous increase in MDA and GSH suggests excessive local ROS production induced by Mn_2_O_3_ nanoparticles, with defense mechanisms mobilized but insufficient, as indicated by increased CASP-3 activation and initiation of apoptotic pathways in intestinal cells [32,45].

In the brains of rats supplemented with Mn_2_O_3_ nanoparticles, increased levels of 8-OHdG, CASP-3, CASP-8, and 3-NT were accompanied by decreased GSH and PC contents. This alarming profile indicates pronounced neurotoxicity. The observed neurotoxicity profile suggests Mn_2_O_3_ nanoparticles may reach brain tissue via blood–brain barrier penetration [23]. Depletion of GSH suggests complete exhaustion of the primary non-enzymatic antioxidant, while increased 8-OHdG, CASP-3, CASP-8, and 3-NT indicate extensive DNA damage, intense protein nitration, and activation of apoptotic pathways in brain homogenates, potentially involving neuronal and glial cells.

The paradoxical reduction in brain PC levels, despite extensive oxidative DNA damage and apoptosis, likely reflects massive neuronal cytolysis rather than protection. Mn_2_O_3_ NPs-induced OH generation via redox cycling triggers rapid necrosis and lysis of vulnerable neurons, releasing carbonylated proteins extracellularly into CSF/gliosis spaces. This pharmacological consequence, the depletion of intracellular proteome with extracellular protein debris accumulation, may promote secondary neuroinflammation via microglial activation and exacerbate long-term neurodegeneration, mirroring manganism pathology. Consequently, Mn_2_O_3_ NPs exhibit acute cytotoxicity followed by chronic inflammatory sequelae, underscoring their unsuitability as dietary supplements [21,26,27]. Manganese is known to cross the blood–brain barrier and act as a pro-oxidant in the central nervous system; nanoparticle formulations may exacerbate this toxicity through enhanced bioavailability and localized deposition, particularly within the basal ganglia [23,25].

The present findings demonstrate that higher plasma levels of GSH and Zn and lower renal CASP-8 content in rats fed Mn_2_O_3_ nanoparticles, compared with animals subjected to complete Mn deficiency, indicate substantial differences between these two interventions. Importantly, Mn deficiency induced tissue-specific oxidative stress patterns: decreased PC in plasma and brain, but elevated PC in the liver and jejunum. This organ selectivity reflects differential antioxidant capacity and Mn-SOD dependence, with plasma/brain showing rapid PC depletion due to proteasomal clearance and limited reserves, while liver/jejunum exhibit persistent protein oxidation due to high metabolic demands. Immunological analyses and gut–brain axis investigations from our complementary studies [31,32], as discussed above, support these biochemical findings of organ-specific oxidative stress and toxicity patterns. While total Mn deficiency induces adverse changes in the brain and intestine, replacement of the standard manganese form with nanoparticles provides selective benefits in the liver and kidneys, albeit at the cost of neurotoxicity. Given the superior bioavailability of Mn_2_O_3_ NPs compared to MnCO_3_, the standard dose of 65 mg/kg may exceed the therapeutic window, causing neurotoxicity while hepatic/renal benefits plateau. Dose–response studies using lower NPs doses are essential to determine if hepatorenal protection can be achieved without brain toxicity, potentially identifying an optimal supplementation strategy [29].

The dual beneficial/adverse effects of both Mn deficiency and Mn_2_O_3_ nanoparticles arise from tissue-specific Mn-SOD dependence, bioavailability differences, and metabolic demands. Mn deficiency impairs high-Mn-SOD tissues (brain, plasma) while sparing/adapting others (liver, kidney), whereas nanoparticles enhance hepatic/renal uptake but exacerbate neurotoxicity via blood–brain barrier penetration and redox cycling. This organ selectivity underscores Mn’s narrow therapeutic window and nanoparticle-specific pharmacokinetics. Both manganese deficiency and excess can adversely affect immune function and promote oxidative stress [17,19]. The observed differences between the Mn_2_O_3_ nanoparticle-supplemented group and the control group suggest that despite potential advantages related to bioavailability and biological efficiency, manganese nanoparticles may pose a significant risk of organ-specific toxicity, particularly within the central nervous system. Future studies should examine Nrf2 signaling to elucidate upstream regulatory mechanisms of these tissue-specific responses.

Wistar rats, as a recognized model of manganese toxicodynamics, enable the assessment of nanoparticle effects in small laboratory animals. However, direct translation of results to humans or farm animals (e.g., pigs, poultry) requires caution due to differences in metabolism and exposure time. The effects of Mn on rat redox status mirror human physiology, confirming its relevance for neurotoxicity risk assessment; however, the human dietary requirement for Mn (~4.5 mg/day) and longer lifespan require dose adjustments and chronic studies.

## 5. Limitations

Despite the comprehensive biochemical profiling across multiple organs, several limitations should be considered when interpreting these results. Direct tissue Mn quantification across organs was not performed, limiting precise correlation between local Mn concentrations and observed toxicity patterns. Direct histological examination of liver, kidney, brain, and jejunum tissues was not performed, limiting morphological confirmation of the observed oxidative, nitrosative, and apoptotic damage patterns. The 12-week experimental duration captures acute-to-subchronic effects but may not reflect chronic, lifespan exposure scenarios, where neurotoxic effects such as GSH depletion and DNA damage could either plateau through adaptation or escalate progressively toward irreversible neurodegeneration. Both MnCO_3_ and Mn_2_O_3_ nanoparticles were administered at the standard 65 mg/kg dose; given nanoparticles’ superior bioavailability, this fixed dose likely exceeds the therapeutic window, potentially causing neurotoxicity while hepatic/renal benefits may plateau at lower concentrations. Functional outcomes including neurobehavioral testing, renal clearance, and intestinal permeability assessments were not conducted to correlate molecular changes with physiological impairment. These limitations highlight critical directions for future research including chronic exposure studies, dose optimization, comprehensive histopathology, tissue Mn analysis, and functional assessments.

## 6. Conclusions

The conducted studies demonstrated that complete exclusion of manganese from the diet leads to organ-specific dysregulation of antioxidant defense systems. The liver activates effective compensatory mechanisms that protect DNA integrity, albeit at the cost of increased oxidative protein damage. The kidneys rely on alternative antioxidant systems, whereas the intestine and brain undergo rapid exhaustion of antioxidant defenses and extensive oxidative–nitrosative damage, which may have long-term health consequences.

Replacement of the conventional manganese form (MnCO_3_) with Mn_2_O_3_ nanoparticles (Mn_2_O_3_NPs) resulted in a shift in systemic antioxidant balance. In plasma and liver, nanoparticles increased GSH levels, total antioxidant status (TAS), and Mn-SOD activity, indicating enhanced overall antioxidant capacity and reduced nitrosative stress. Simultaneously, increased markers of oxidative protein damage (PC) and intensified apoptosis (CASP-8) were observed, particularly in the intestine and brain. These findings suggest that although Mn_2_O_3_ nanoparticles improve manganese bioavailability and exert protective effects in the liver and kidneys, their impact on the central nervous system and intestine promotes enhanced neurotoxicity and apoptosis. This organ-specific toxicity indicates that manganese (III) oxide nanoparticles should not be considered a safe form of dietary manganese supplementation at the tested dose and regimen.

## Figures and Tables

**Table 4:** Indicators of oxidoreductive status in rat blood (n = 8 per group).

	Control (K)	Nano-Mn (N)	Without Mn (B)	SEM	*p*-Value
3-NT, ng/mL	18.40 ± 2.23	18.02 ± 1.81	19.04 ± 1.00	0.35	0.284
SOD, ng/mL	3.32 ± 0.84 ^a^	2.27 ± 0.69 ^b^	2.18 ± 0.54 ^b^	0.17	0.005
MDA, nmol/mL	1.38 ± 0.24 ^b^	1.31 ± 0.20 ^b^	1.71 ± 0.21 ^a^	0.06	0.002
CAT, ng/mL	30.94 ± 1.57	31.62 ± 3.01	32.03 ± 3.80	0.58	0.492
8-OHdG, ng/mL	1.72 ± 0.27	1.96 ± 0.27	1.96 ± 0.20	0.05	0.067
CASP-3, ng/mL	1.22 ± 0.22	1.34 ± 0.04	1.32 ± 0.21	0.04	0.181
CASP-8, ng/mL	2.55 ± 0.25 ^b^	3.27 ± 0.38 ^a^	3.60 ± 0.57 ^a^	0.12	<0.001
TAS, mmol/L	1.39 ± 0.40 ^b^	1.81 ± 0.22 ^a^	1.65 ± 0.23 ^ab^	0.07	0.013
TGSH, µmol/L	4.78 ± 0.20	4.77 ± 0.27	4.60 ± 0.10	0.04	0.097
GSSG, µmol/L	0.74 ± 0.20	0.59 ± 0.10	0.69 ± 0.15	0.03	0.077
GSH, µmol/L	3.31 ± 0.30 ^ab^	3.59 ± 0.33 ^a^	3.22 ± 0.27 ^b^	0.07	0.027
PC, nmol/mg protein	2.87 ± 0.56 ^a^	2.43 ± 0.64 ^a^	1.85 ± 0.28 ^b^	0.13	0.001
%-mC	0.51 ± 0.18	0.65 ± 0.14	0.67 ± 0.18	0.04	0.088
Fe, µmol/L	73.75 ± 49.61	69.06 ± 46.31	61.41 ± 32.16	8.52	0.597
Cu, µmol/L	21.23 ± 10.88 ^a^	12.35 ± 4.19 ^b^	24.80 ± 6.83 ^a^	1.87	0.006
Zn, µmol/L	132.00 ± 9.48 ^ab^	150.00 ± 22.43 ^a^	122.00 ± 24.51 ^b^	4.59	0.013

SEM, pooled standard error of mean (standard deviation for all rats divided by the square root of rat number, n = 24); ^a,b^ mean values within a row with unlike superscript letters are shown to be significantly different (*p* < 0.05).

**Table 5:** Indicators of oxidoreductive status and protein nitration in rat liver (n = 8 per group).

	Control (K)	Nano-Mn (N)	Without Mn (B)	SEM	*p*-Value
MDA (µmol/g)	0.025 ± 0.004	0.026 ± 0.004	0.023 ± 0.003	0.001	0.240
SOD (U/g)	468.60 ± 108.16 ^b^	772.60 ± 87.00 ^a^	838.30 ± 108.16 ^a^	38.99	<0.001
CAT (U/g)	2322.00 ± 292.76	2240.00 ± 254.75	2224.00 ± 238.24	52.12	0.727
GSH (µmol/g)	0.15 ± 0.04 ^b^	0.16 ± 0.05 ^b^	0.20 ± 0.02 ^a^	0.01	0.016
8-OHdG, ng/g	5.24 ± 1.29 ^a^	5.79 ± 0.90 ^a^	4.14 ± 0.62 ^b^	0.24	0.004
CASP-3, ng/g	288.00 ± 27.42	311.00 ± 74.13	301.00 ± 75.88	12.50	0.499
CASP-8, ng/g	107.00 ± 34.92	85.54 ± 23.55	78.34 ± 19.39	5.80	0.056
3-NT, ng/g	369.00 ± 79.56 ^a^	291.00 ± 56.49 ^b^	227.00 ± 51.78 ^b^	17.40	<0.001
PC, nmol/mg protein	0.06 ± 0.01 ^b^	0.08 ± 0.01 ^a^	0.09 ± 0.02 ^a^	0.003	0.005

SEM, pooled standard error of mean (standard deviation for all rats divided by the square root of rat number, n = 24); ^a,b^ mean values within a row with unlike superscript letters are shown to be significantly different (*p* < 0.05).

**Table 6:** Indicators of oxidoreductive status and protein nitration in rat kidneys (n = 8 per group).

	Control (K)	Nano-Mn (N)	Without Mn (B)	SEM	*p*-Value
MDA (µmol/g)	0.021 ± 0.006 ^a^	0.012 ± 0.003 ^b^	0.019 ± 0.004 ^a^	0.001	0.002
SOD (U/g)	671.60 ± 10.54	662.8 ± 59.233	660.9 ± 11.505	6.97	0.812
CAT (U/g)	730.10 ± 31.76 ^a^	623.1 ± 62.402 ^b^	550.7 ± 134.07 ^b^	22.94	0.002
GSH (µmol/g)	0.009 ± 0.003	0.007 ± 0.003	0.007 ± 0.003	0.001	0.301
8-OHdG, ng/g	2.10 ± 0.18 ^a^	1.94 ± 0.191 ^b^	0.316 ± 0.084 ^c^	0.17	<0.001
CASP-3, ng/g	376.00 ± 77.54	364 ± 8.771	331 ± 59.682	11.75	0.146
CASP-8, ng/g	82.94 ± 11.18 ^ab^	72.41 ± 7.670 ^b^	89.16 ± 11.168 ^a^	2.45	0.005
3-NT, ng/g	270.00 ± 41.73	298 ± 77.841	296 ± 32.938	10.95	0.339
PC, nmol/mg protein	0.086 ± 0.015	0.087 ± 0.016	0.093 ± 0.026	0.004	0.515

SEM, pooled standard error of mean (standard deviation for all rats divided by the square root of rat number, n = 24); ^a–c^ mean values within a row with unlike superscript letters are shown to be significantly different (*p* < 0.05).

**Table 7:** Indicators of oxidoreductive status and protein nitration in the jejunum of rats (n = 8 per group).

	Control (K)	Nano-Mn (N)	Without Mn (B)	SEM	*p*-Value
MDA (µmol/g)	0.027 ± 0.006 ^b^	0.035 ± 0.004 ^a^	0.034 ± 0.003 ^a^	0.001	0.003
SOD (U/g)	172.60 ± 24.72 ^b^	175.00 ± 22.67 ^b^	247.80 ± 38.46 ^a^	9.27	<0.001
CAT (U/g)	390.80 ± 81.72 ^b^	315.20 ± 83.73 ^b^	686.70 ± 93.02 ^a^	37.42	<0.001
GSH (µmol/g)	0.06 ± 0.02 ^b^	0.08 ± 0.01 ^a^	0.07 ± 0.02 ^ab^	0.003	0.046
8-OHdG, ng/g	14.92 ± 1.90	14.14 ± 3.72	16.15 ± 2.51	0.58	0.189
CASP-3, ng/g	6.60 ± 1.31 ^c^	8.43 ± 0.90 ^b^	10.56 ± 1.02 ^a^	0.40	<0.001
CASP-8, ng/g	28.41 ± 7.79	26.28 ± 8.37	34.93 ± 8.28	1.77	0.056
3-NT, ng/g	183.00 ± 50.37 ^b^	191.00 ± 29.52 ^b^	270.00 ± 48.11 ^a^	11.82	<0.001
PC, nmol/mg protein	0.19 ± 0.07 ^b^	0.15 ± 0.03 ^b^	0.38 ± 0.06 ^a^	0.02	<0.001

SEM, pooled standard error of mean (standard deviation for all rats divided by the square root of rat number, n = 24); ^a–c^ mean values within a row with unlike superscript letters are shown to be significantly different (*p* < 0.05).

**Table 8:** Indicators of oxidoreductive status and protein nitration in the rat brain (n = 8 per group).

	Control (K)	Nano-Mn (N)	Without Mn (B)	SEM	*p*-Value
MDA (µmol/g)	0.039 ± 0.007 ^a^	0.040 ± 0.007 ^a^	0.018 ± 0.001 ^b^	0.002	<0.001
SOD (U/g)	220.70 ± 6.17	231.90 ± 16.93	227.00 ± 12.47	2.65	0.228
CAT (U/g)	175.80 ± 45.52	219.90 ± 20.34	201.60 ± 54.34	9.12	0.139
GSH (µmol/g)	0.021 ± 0.005 ^a^	0.007 ± 0.006 ^b^	0.010 ± 0.002 ^b^	0.002	<0.001
8-OHdG, ng/g	12.36 ± 4.31 ^c^	30.43 ± 2.45 ^a^	20.74 ± 3.86 ^b^	11.70	<0.001
CASP-3, ng/g	16.68 ± 1.38 ^b^	18.54 ± 1.21 ^a^	18.37 ± 1.40 ^a^	0.31	0.014
CASP-8, ng/g	39.86 ± 1.81 ^b^	49.35 ± 8.57 ^a^	42.06 ± 2.20 ^a^	1.32	0.002
3-NT, ng/g	234.00 ± 31.61 ^b^	269.00 ± 34.61 ^a^	270.00 ± 18.92 ^a^	6.66	0.027
PC, nmol/mg protein	0.259 ± 0.023 ^a^	0.213 ± 0.036 ^b^	0.207 ± 0.035 ^b^	0.01	0.006

SEM, pooled standard error of mean (standard deviation for all rats divided by the square root of rat number, n = 24); ^a–c^ mean values within a row with unlike superscript letters are shown to be significantly different (*p* < 0.05).

## Data Availability

Data are contained within the article.
